# Ontological Disintegration and Psychiatric Vulnerability During Pandemics: Ethical Frameworks and Lived Realities

**DOI:** 10.1192/j.eurpsy.2026.11892

**Published:** 2026-07-31

**Authors:** C. Lazzari

**Affiliations:** Psychiatry, International Centre for Healthcare and Medical Education (ICHME), London, United Kingdom

## Abstract

**Introduction:**

Pandemics disrupt not only biological systems but also the foundations of identity, perception, and meaning. COVID-19 exposed the fragility of traditional knowledge structures and evidenced the psychiatric consequences of ontological uncertainty. This study positions pandemics as existential crises requiring philosophical engagement to understand their impact on mental health fully.

**Objectives:**

To examine how pandemic-induced ontological instability manifests in psychiatric contexts, and how ethical interpretation—through religious and secular frameworks—shapes clinical understanding and therapeutic response.

**Methods:**

A combined philosophical–clinical methodology was employed. Drawing on process ontology, pragmatism, and phenomenology, the study included an analysis of a documented case report involving reactive psychosis triggered by COVID-19-related existential distress. The focus was placed on disrupted identity, delusional patterns, and the collapse of relational meaning.

**Results:**

The case involved a 50-year-old male presenting with paranoid delusions and identity confusion during the pandemic, including beliefs in impostor family members and conspiracy-driven narratives. Diagnosed with acute paranoid reaction, the patient responded to antipsychotic treatment. The episode illustrates ontological breakdown as a psychiatric event, conditioned by fear, uncertainty, and epistemic instability.

**Image 1:**
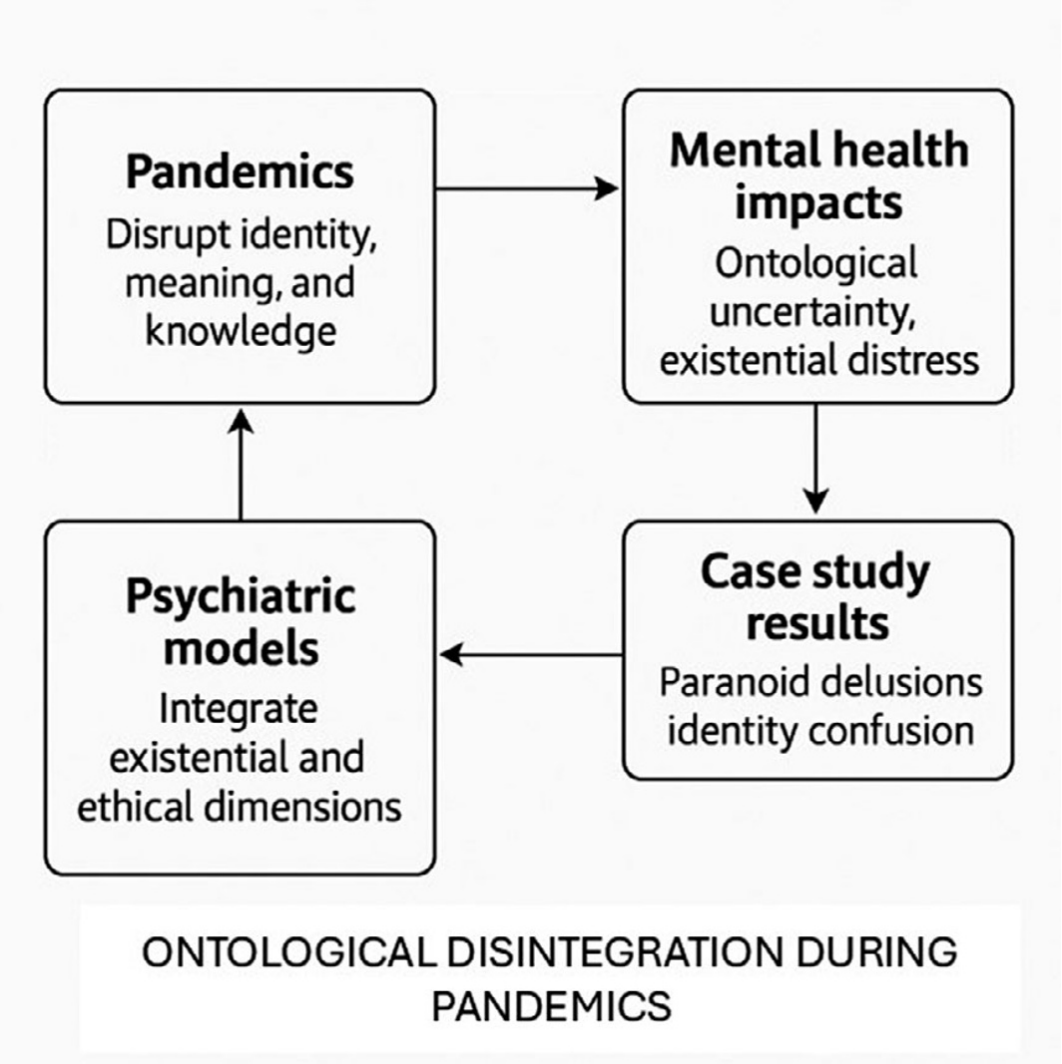
Image 1: Long description.

**Conclusions:**

Pandemics provoke ontological and psychological disintegration, especially in vulnerable individuals. Psychiatric models that integrate existential, phenomenological, and ethical dimensions are essential to address the depth of crisis-induced mental health challenges. Philosophical engagement offers critical insight into how reality, identity, and institutional dynamics interact in times of collective disruption.

**Disclosure of Interest:**

None Declared

## Long descriptions

### Image 1: Long description

The flowchart consists of four rectangular boxes arranged in a square formation with arrows indicating a clockwise flow.

1. Top-left node: Pandemics. Text below states: Disrupt identity, meaning, and knowledge. An arrow points right to the next node.

2. Top-right node: Mental health impacts. Text below states: Ontological uncertainty, existential distress. An arrow points down to the next node.

3. Bottom-right node: Case study results. Text below states: Paranoid delusions identity confusion. An arrow points left to the next node.

4. Bottom-left node: Psychiatric models. Text below states: Integrate existential and ethical dimensions. An arrow points up, returning to the first node to complete the cycle.

Centered at the bottom of the diagram is the title: ONTOLOGICAL DISINTEGRATION DURING PANDEMICS.

Navigate back to Image 1:.

